# The perceptions of European geriatricians on the co-occurrence and links between dementia, delirium and frailty

**DOI:** 10.1007/s41999-025-01173-4

**Published:** 2025-03-15

**Authors:** Mary Faherty, Catriona Curtin, Giuseppe Bellelli, Enrico Brunetti, Mario Bo, Alessandro Morandi, Antonio Cherubini, Massimiliano Fedecostante, Maria Cristina Ferrara, Alessandra Coin, Susan D. Shenkin, Pinar Soysal, Suzanne Timmons

**Affiliations:** 1 Centre for Gerontology and Rehabilitation, University College Cork, Cork, Ireland; 2 School of Medicina and Surgery, University of Milano-Bicocca and Acute Geriatric Unit, IRCCS Foundation “San Gerardo Dei Tintori”, Monza, Italy; 3 Section of Geriatrics, Department of Medical Sciences, University Hospital Città della Salute e della Scienza, Molinette, Turin, Italy; 4 Department of Experimental and Clinical Medicine, University of Florence, Florence, Italy; 5 Department of Clinical and Experimental Science, University of Brescia, Brescia, Italy; 6 Azienda Speciale Cremona Solidale, Cremona, Italy; 7 Vall d’Hebrón Institute of Research, Barcelona, Spain; 8 Accettazione Geriatrica e Centro di Ricerca per l’Invecchiamento, IRCCS INRCA, Ancona, Italy; 9 Department of Clinical and Molecular Sciences, Università Politecnica delle Marche, Ancona, Italy; 10 School of Medicina and Surgery, University of Milano-Bicocca, Milan, Italy; 11 Geriatrics Unit, Department of Medicine, Azienda Ospedale—Università Padua, University of Padova, Padova, Italy; 12 Ageing and Health Research Group, Usher Institute, University of Edinburgh, Edinburgh, Scotland; 13 Department of Geriatric Medicine, Faculty of Medicine, Bezmialem Vakif University, Istanbul, Türkiye

**Keywords:** Delirium, Dementia, Frailty, Attitudes, Risk factors, Prevalence

## Abstract

**Purpose:**

This study aimed to explore the perceptions of geriatricians and experienced geriatric trainees in Europe of the complex relationship between dementia, delirium and frailty, including their relative prevalence, overlaps and causality.

**Methods:**

An online anonymous survey was administered across 30 European countries, via the mailing list of the European Geriatric Medicine Society (EuGMS), national member groups and the authors’ professional networks. Questions were framed in the context of recently hospitalised 80-year-old patients.

**Results:**

Within the 440 included surveys, respondents particularly over-estimated frailty prevalence in older hospitalised patients, with two-thirds choosing between 41 and 80% prevalence, when the literature suggests only 23–46%, but paradoxically underestimated the frequency of frailty in people with delirium (more than three quarters of responses across 21–80%; literature suggests 71–93%). Severe dementia and previous delirium were correctly considered the strongest risks for future inpatient delirium. However, many considered pre-frailty a moderate (44%) or even strong (19%) risk for future delirium, while a minority considered severe dementia a low risk. Respondents viewed delirium superimposed on dementia (DSD) as having the strongest influence on in-hospital mortality and discharge to residential care, dementia as having the strongest influence on future residential care admission, and frailty as the condition most strongly influencing future mortality.

**Conclusion:**

Geriatricians and experienced geriatric trainees across Europe gave varied responses to questions about delirium, dementia and frailty prevalence, co-occurrence and consequences. This indicates a need for the performance and wide dissemination of robust, prospective research examining all three conditions in older hospital cohorts. We suggest a merging of selective delirium-frailty and dementia-delirium interests to the dementia-delirium-frailty triumvirate.

**Supplementary Information:**

The online version contains supplementary material available at 10.1007/s41999-025-01173-4.

## Key summary points

**Aim:**

To explore the perceptions of geriatricians and experienced geriatric trainees in Europe on the complex relationships between dementia, delirium and frailty.

**Findings:**

European geriatricians overestimate the prevalence of frailty and to a lesser extent delirium in older hospitalised adults, while underestimating the probability of older inpatients with delirium also having frailty. As expected, severe dementia and prior delirium were rated as the strongest risks for future delirium, but more than half the cohort considered pre-frailty a moderate or strong risk factor.

**Message:**

Research with a dementia, delirium or frailty focus needs to carefully determine the presence and influence of the two other conditions to give more rounded and real-life data that can better inform education and clinical practice around screening and prognostication.

**Supplementary Information:**

The online version contains supplementary material available at 10.1007/s41999-025-01173-4.

## Introduction

Health and social care provision for older people can be hindered by siloed thinking, when disease states are considered in isolation instead of taking a holistic approach. In reality, many disease states in older people coexist and overlap, with common causality, and/or shared symptoms, and/or additive or even multiplicative effects.

Delirium and dementia have well established links in older people, with dementia recognised as a key risk factor for delirium [1, 2] and delirium recognised as a risk factor for dementia [3, 4]. Studies show dementia prevalence rates for hospital inpatients of 26–63% (mean age 80) [5]. Delirium, a severe neuropsychiatric condition characterised by an acute and fluctuating disturbance in attention and awareness [6], has an estimated prevalence in older people with dementia in hospital settings of 49–57% [7–9]. Delirium can not only unmask mild, previously unrecognised dementia [10] but can also precipitate and accelerate dementia, as shown in epidemiological studies [4, 11, 12].

Frailty is a condition, related to the ageing process, characterised by reduced physiological reserves [13] and its relationship to dementia is well recognised [14–17]. Both syndromes share a common risk factor: older age. Frailty, if considered across all domains of the human condition, naturally aligns to dementia [18]. There are dozens of tools for measuring frailty. Fried’s phenotype model focuses on physical frailty, excluding cognitive and psychosocial features [19]. Thus, a person with dementia will not have frailty under this model until the dementia is complicated by physical frailty, or the person develops other conditions that cause frailty. The nine-point Rockwood Clinical Frailty Scale (CFS), based on the cumulative deficit model, takes a simplistic approach to frailty-dementia, whereby the degree of dementia corresponds to the degree of frailty [20]. The more detailed Frailty Index quantifies deficits into a coefficient score for the “whole organism” as opposed to a particular functional deficiency or decline [[21], p.332]. Not surprisingly, frailty rates vary depending on the diagnostic tool used; using the Frailty Index gives higher prevalence figures than the phenotype model [18, 22]. On the co-occurrence of frailty and dementia, a population-based study using the phenotype model found frail people, of average age 82, were eight times more likely to have dementia than robust people [23].

The link between frailty and delirium is complex. These two syndromes are frequently grouped together due to their commonalities: both relate to ageing, have poor clinical outcomes, influence each other and share a common pathophysiology of inflamm-ageing and immune-senescence [24]. Vulnerability to factors including age, genetics, pathology and environment may cause the clinical expressions of frailty and delirium [17]. However, while frailty is chronic and characterised by decreased functional reserves, including muscular weakness, delirium is acute and the primary dysfunction is cognitive, although delirium of course also affects motor behaviour and control [24]. Despite this, frailty and delirium are often presented together, the premise being that frail older people are at greater risk of delirium, which is indeed true [21, 25]. They are also more likely to have a missed delirium diagnosis [9]. A recent SR and MA of hospitalised older adults (2022) found a delirium relative risk (RR) of 1.66 in those with frailty [26], while an SR and MA in 2021 found a RR of 2.96 [21]. However, this increased risk may be due to indirect associations, wherein the greatest risk for delirium is a vulnerable brain, which is often associated with frailty. More recently, the concept of “cognitive frailty” has been proposed as the combination of physical frailty and mild cognitive impairment (MCI) [27]. It is not clear if this definition is helpful, as clearly dementia rather than MCI is the ultimate “cognitive frailty” if this is viewed in terms of cognitive vulnerability.

Given these complex relationships, and the varying risk and prevalence of each condition depending on the constructs being considered, this study aims to explore how geriatricians in Europe understand the concepts of frailty, dementia and delirium. In particular, we wished to consider perceptions of linkages, causality directions and magnitude, and the relative impact of the three conditions on outcomes of an older person admitted to hospital.

## Methods

### Study design

A quantitative design was employed, with participants completing an anonymous online survey, with an estimated completion time of 6–12 min. To encourage participants to proceed to the end of the survey, they were given the option to skip some questions. Participation in this study was completely voluntary.

### Sampling and recruitment

The survey was open to geriatricians and geriatrician trainees working in Europe. Participants were eligible if they satisfied one of the following three criteria: (i) fully qualified consultant geriatrician (temporary or permanent) working in a European country (currently or within the last 12 months); (ii) geriatric trainee in their final two years of specialist (higher) geriatric training in any European country and (iii) geriatrician retired within the previous three years from work in any European country. For countries where geriatric medicine is not a stand-alone speciality, a general physician with a special interest in geriatric medicine was eligible. There were no restrictions based on age or gender, and geriatricians did not need to be particularly attuned to delirium, dementia or frailty in their clinical practice to participate in the survey.

Participants were recruited using snowball sampling. The survey was sent to all members of the European Geriatric Medicine Society (EuGMS), with members encouraged to share the link with colleagues and experienced trainees (who may not be EuGMS members). The survey was also promoted by members of the EuGMS Special Interest Groups in Dementia, Delirium and Frailty through their national geriatric professional bodies.

### Data collection and analysis

Data were collected between September 2023 and June 2024 using an online Qualtrics survey form. The survey was created de novo to answer the research questions, by members of the EuGMS Special Interest Group (SIG) in Delirium, supported by the SIG in Dementia. It was piloted in English and refined, and then translated into eleven common European languages, available via an embedded link to a project website. Participants were requested to provide answers in English only.

Quantitative data were analysed in Excel, with descriptive statistics presented as the number or percentage of participants endorsing a particular response, and frequency distributions. For survey questions on risk factors for delirium, respondents answered using a scale of 0–10, with 0 indicating no risk and 10 indicating a very strong risk. To simplify the analysis and interpretation of the data, the results were clustered into three approximately equal categories: 7–10 grouped as high risk, 4–6 as moderate risk and 0–3 as low risk. To examine the influence of considering specific frailty models on participants’ estimates of frailty prevalence, an ordinal logistic regression analysis was conducted using the “polr” function from the MASS package in statistical software R.

## Results

### Demographic characteristics of the sample

In total, 536 people opened the survey, of whom 76 deemed themselves ineligible (i.e. they did not satisfy any of the three criteria) and a further 14 did not consent (and therefore did not complete the survey). Six responses from countries outside of Europe (Australia, Israel) were excluded, giving a final sample size of 440 participants. Participants’ employment base, where indicated (*n* = 380), covered 30 European countries (see supplementary material). Participation from Eastern European countries was underrepresented (Table 1).

**Table 1:**
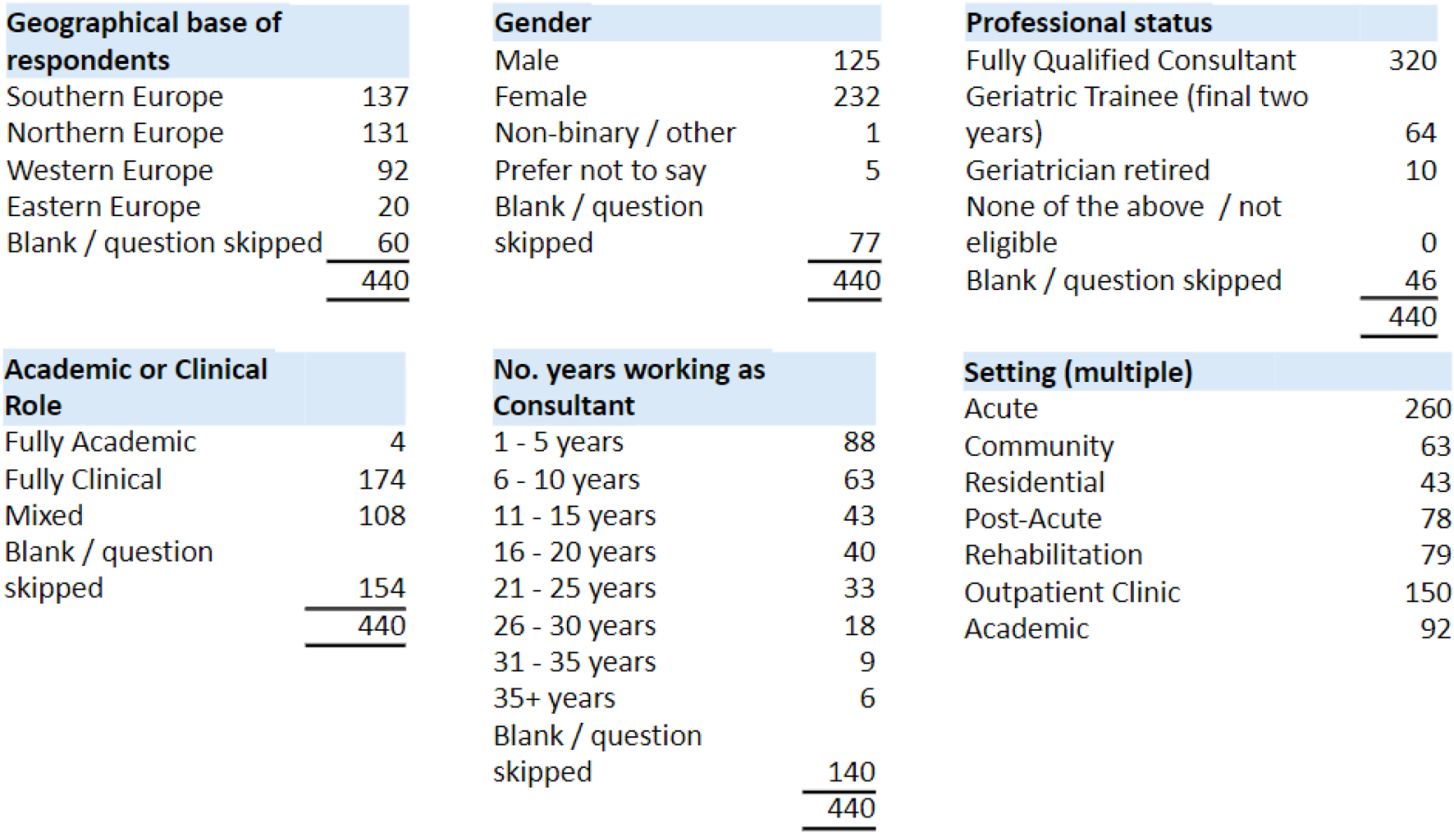
Comparison of various cloaking configurations with different types

Participant responses to the optional question on gender (*n* = 363) indicated that 63.9% were female, 34.4% male, 0.3% non-binary / other, while 1.4% preferred not to say (Table 1). In terms of working role, where indicated, 81.2% were fully qualified consultants (*n* = 320), of whom 294 were in permanent positions; 16.2% were geriatric trainees (*n* = 64) and 2.5% (*n* = 10) were retired geriatricians. Of those who selected a professional categorisation (*n* = 286), 60.8% classified themselves as fully clinical (*n* = 174), 37.8% were a mix of academic and clinical (*n* = 108), while 1.4% were fully academic (*n* = 4). Of the 300 consultants who indicated their experience, 50.3% had 1–10 years’ experience (*n* = 151), 27.7% had 11–20 years’ experience (*n* = 83) and 22% had more than 20 years’ experience (*n* = 66).

Participants’ main special interest areas (as per selected options from the EuGMS SIG categories), in descending order, were dementia; comprehensive geriatric assessment; delirium; frailty and resilience; and falls and fractures. The primary lead roles of participants were, in descending order, in the following areas: dementia; comprehensive geriatric assessment; education and training; delirium; and falls and fractures.

When asked which frailty models or tools they most commonly used (multiple answers allowed), most respondents chose the (Rockwood) Clinical Frailty Scale tool (*n* = 218), with fewer selecting the (Rockwood) Cumulative Deficit / Frailty Index model (*n* = 147) and the Fried phenotype model (*n* = 146).

### Prevalence of delirium, dementia and frailty

**Fig. 1:**
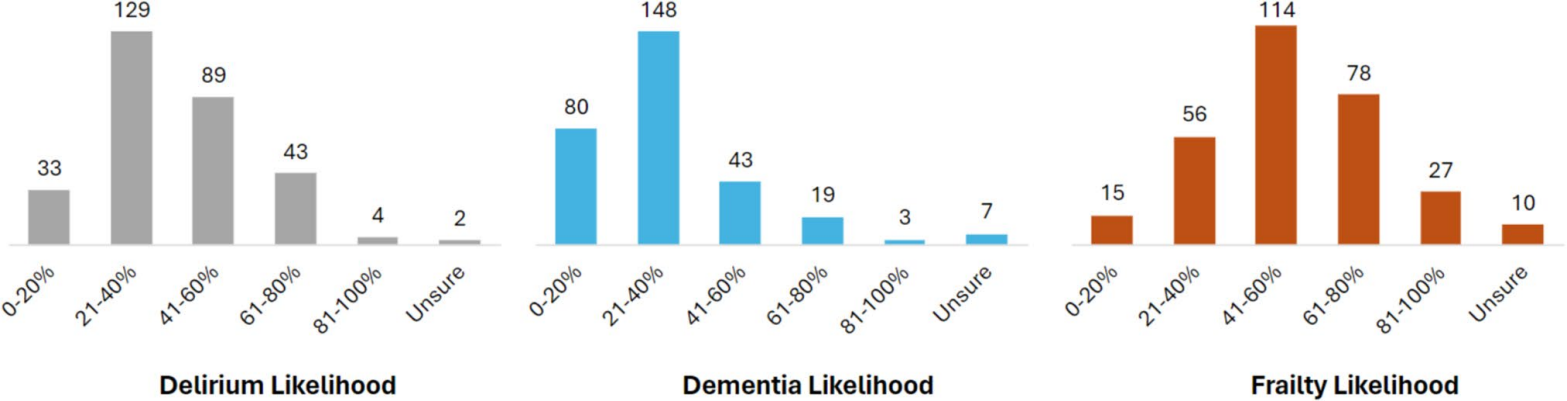
The frequency of selection of the offered “likely prevalence” categories by respondents (category width 20%), for delirium, dementia and frailty, for a hypothesised group of 80-year-olds with an unplanned acute admission within the preceding 48 h

To assess perceived prevalence of delirium, dementia and frailty, participants were asked to consider a group of 80-year-old patients within the first 48 h after an unplanned admission to an acute hospital. For this cohort, participants were asked what percentage were likely to have delirium, dementia or frailty, of any severity, see Fig. 1.

To summarise, dementia was considered the least common condition, peaking at 21–40%, with nine in ten respondents selecting 0–60% prevalence. Delirium was considered more prevalent, with almost two-thirds selecting 21–60% (again peaking at 21–40%).

In contrast, frailty was considered by two-thirds of respondents to occur in 41–80% of cases, with a peak category of 41–60%. Indeed, frailty prevalence had the widest spread of answers, with almost as many selecting “0–40%” as “61–80%” prevalence. In the regression model, those considering the Fried model had a slightly increased perception of higher frailty prevalence (OR of 1.07, CI 0.66–1.7) but this was not statistically significant (*p* = 0.77). Both the Frailty Index (OR of 0.74, CI 0.47–1.2) and CFS (OR of 0.61 (CI 0.37–1.00) showed non-significantly decreased odds of reporting higher prevalence (*p* = 0.19 and *p* = 0.05, respectively). This suggests the frailty model considered by respondents did not affect their frailty prevalence estimates.

### Co-occurrence of delirium, dementia and frailty

**Fig. 2:**
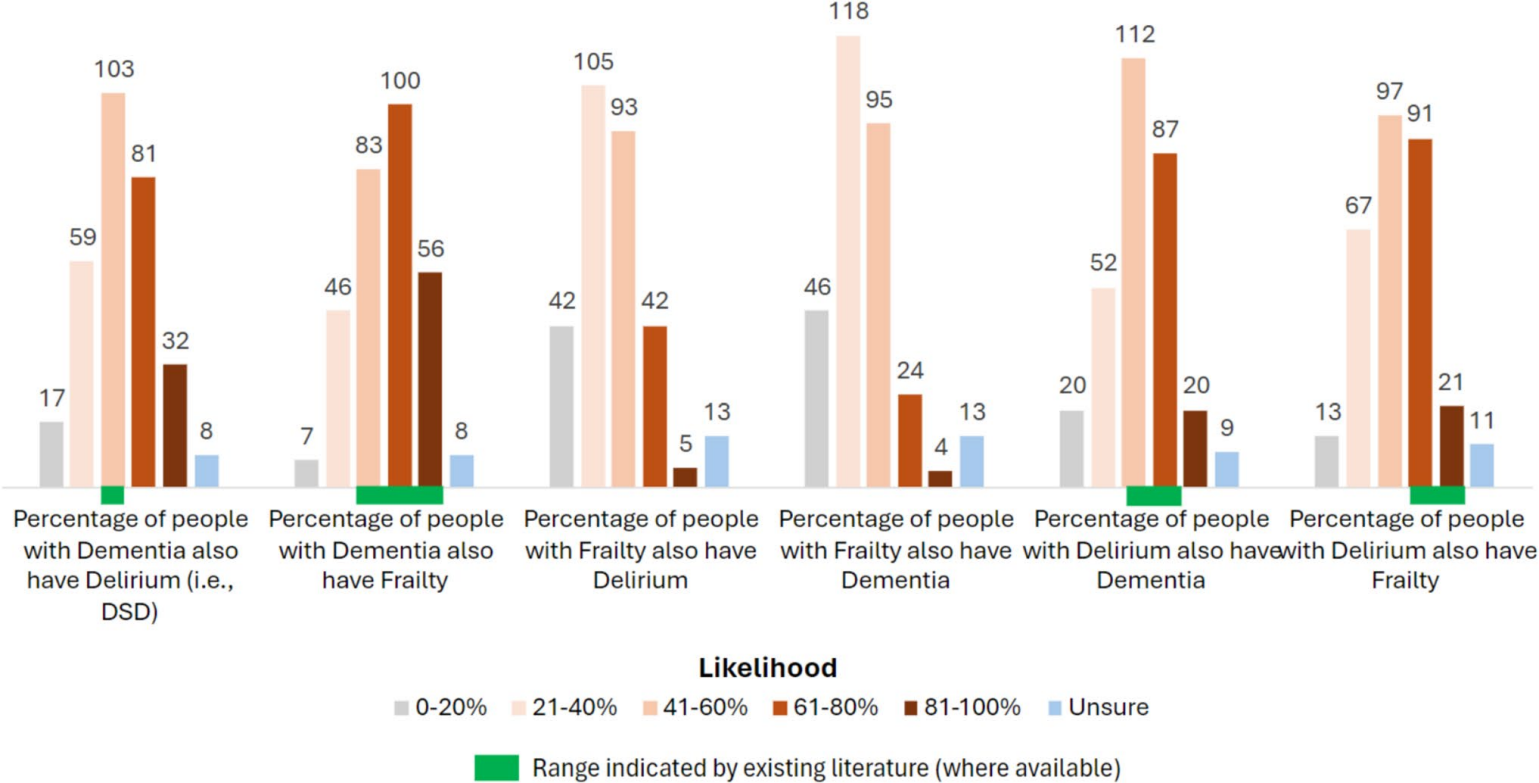
Respondent opinions on co-occurrence of conditions: Percentage of people with dementia who also have delirium (i.e. DSD) or frailty; percentage of people with frailty who also have delirium or dementia; percentage of people with delirium who also have dementia or frailty. Green boxes indicate ranges as indicated by existing literature

To explore the opinion of respondents on the co-occurrence of the three conditions, respondents were asked to select the probability of each condition presenting concurrently with one of the other two conditions, for a group of 80-year-old patients in the first 48 h after an unplanned admission to an acute hospital. Figure 2 summarises the 298 responses.

For older people with dementia in hospital, the peak frequency category chosen by respondents for the person to also have delirium (i.e. delirium superimposed on dementia, DSD) was 41–60%, with most responses between 21 and 80%. For the percentage of people with dementia who would also have frailty, the peak category was higher, at 61–80%, with most responses also spread from 21 to 80%. Thus, dementia-frailty is considered more prevalent than DSD.

For the percentage of older people with frailty in hospital who would also have delirium, most chose the categories of 21–60%, and similarly for the percentage of frail inpatients who would have dementia. Thus, respondents considered people with dementia more often to be frail, than people with frailty to also have dementia.

For the percentage of older people in hospital with delirium who would also have dementia, the most common categories selected were 41–80%, with a peak response of 41–60%. For the percentage of people with delirium who also have frailty, responses were quite evenly spread across the categories covering 21–80%.

### Risk factors for delirium

**Fig. 3:**
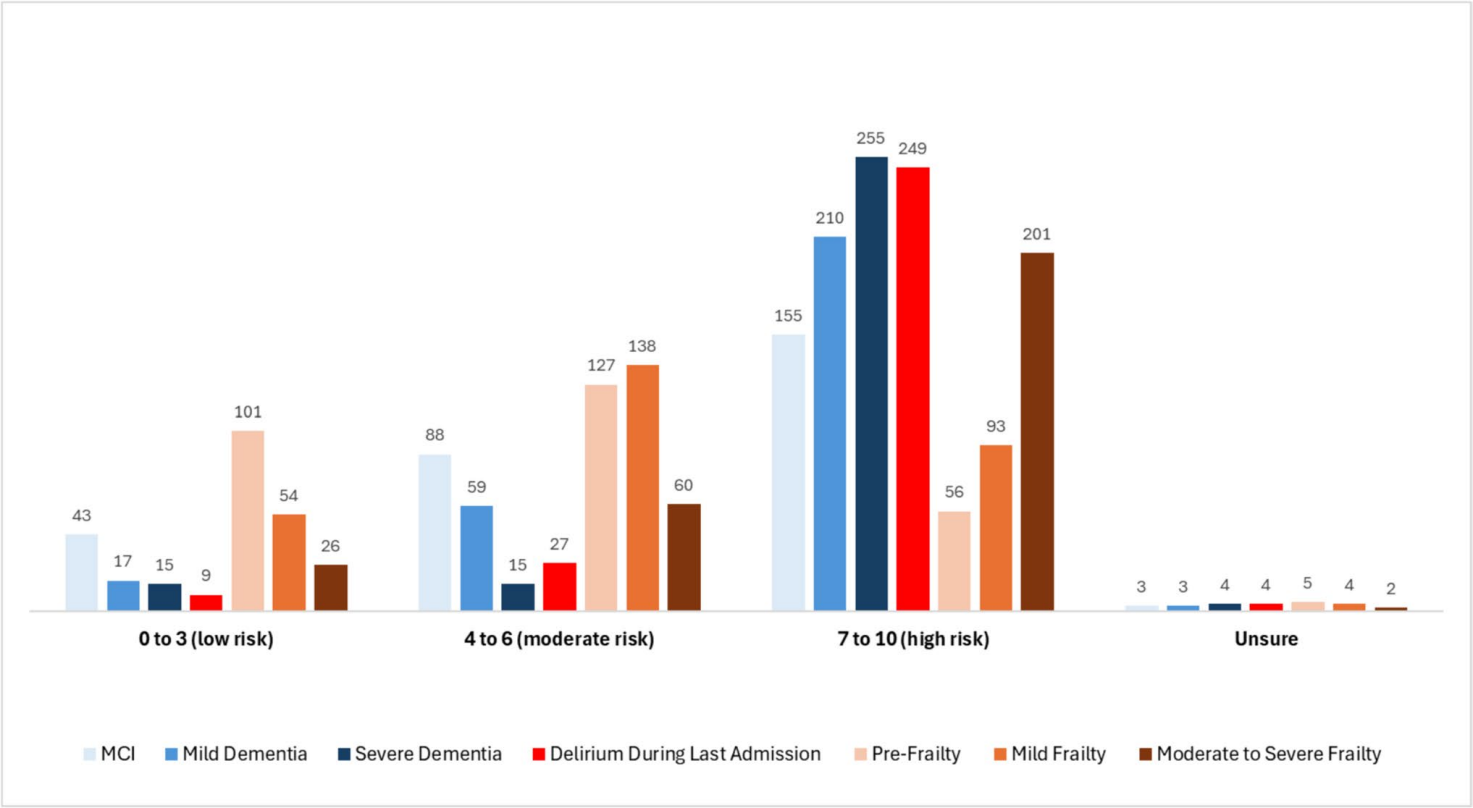
Respondents’ assessment of the strength of selected risk factors for developing incident delirium

To elicit opinions on what risk factors influence the development of incident delirium, respondents were asked to consider an 80-year-old patient admitted to hospital with a urinary tract infection. For this scenario, respondents rated seven risk factors (prior delirium, mild cognitive impairment, and different severities of dementia and frailty). Figure 3 displays the breakdown of the 289 responses (full data in supplementary material).

As expected, respondents identified cognitive impairment as being a significant risk factor for delirium, with a gradient of risk from MCI to severe dementia. Overall, severe dementia was rated as the highest risk factor of all options given to respondents. Thus, 88% of respondents rated severe dementia as being a high-risk factor for incident delirium (*n* = 255) although some rated it a moderate or low risk factor (*n* = 15 for both options), while 4 people were unsure. Mild dementia was rated as a high-risk factor by 73% (*n* = 210), while 20% viewed it as a moderate risk factor (*n* = 59) and 6% rated it a low risk factor (*n* = 17). And finally, 54% of respondents rated MCI as high risk (*n* = 155), 30% rated it a moderate risk (*n* = 88) and 15% scored it a low risk factor (*n* = 43). As expected, delirium during a previous admission was rated by 86% as being a high-risk factor (*n* = 249), although 9% rated it only a moderate risk factor (*n* = 27) and 3% rated it a low risk factor (*n* = 9) with 1% unsure (*n* = 4).

There was a similar graded risk for frailty, with 70% considering moderate to severe frailty to be a high-risk factor for incident delirium (*n* = 201), 21% a moderate risk factor (*n* = 60) and 9% a low risk factor (*n* = 26). Both pre-frailty and mild frailty had lower scores. 44% of respondents considered pre-frailty a moderate risk (*n* = 127), while it was rated as a low or high-risk factor by 35% (*n* = 101) and 19% (*n* = 56), respectively. Similarly, almost half (48%) scored mild frailty as a moderate risk (*n* = 138), although 32% (*n* = 93) rated it a high-risk factor, with 19% rating it a low risk factor (*n* = 54).

To summarise, severe dementia and previous delirium were identified as high-risk states for incident delirium, while moderate to severe frailty was seen as having almost a similar risk for delirium as mild dementia, and indeed a higher risk than MCI, which in turn was considered a higher risk than mild frailty or pre-frailty.

Respondents were also asked if delirium psychomotor subtypes (e.g. hypoactive, hyperactive, etc.) affected the risk of developing a future delirium episode. The 289 responses showed no consensus of opinion, with 29% considering psychomotor type a strong influence (*n* = 85), 31% a moderate influence (*n* = 88) and 27% a minor influence (*n* = 78), while 13% were unsure (*n* = 38).

### Significance of delirium, dementia, frailty and DSD for prognosis

**Fig. 4:**
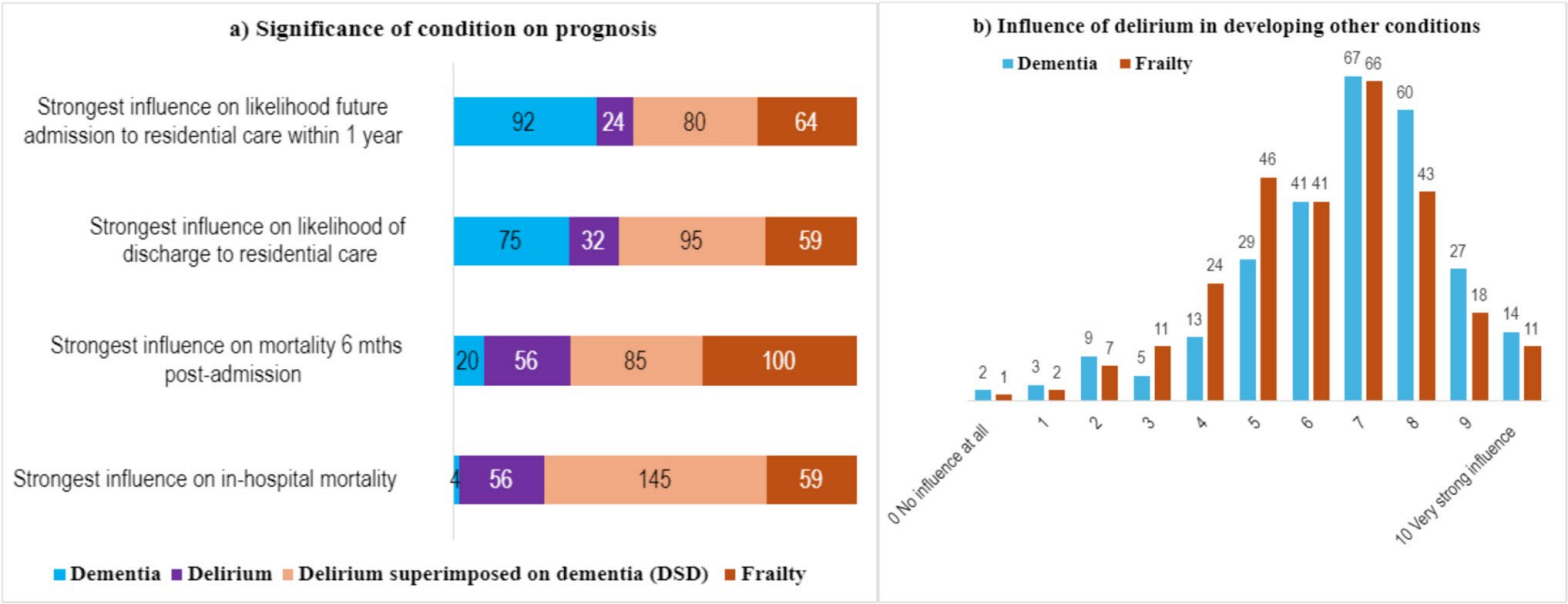
Respondents’ perceptions of (**a**) the significance of dementia, delirium, DSD and frailty for prognosis in terms of later admission or immediate discharge to residential care, or 6-month or in-hospital mortality (counts shown); **b** influence of episode of delirium on the future development of dementia or frailty

Participants were asked four questions to determine the prognostic significance of four conditions (dementia, frailty, delirium, DSD) for prognosis, based on the scenario of an 80-year-old patient admitted to hospital with a urinary tract infection. Figure 4a) outlines the findings.

In terms of the strongest influence on *in-hospital mortality* (*n* = 272 replies), more than half of respondents (53%) selected DSD, while 22% selected frailty and 21% delirium alone. Dementia was only selected by 4 respondents. DSD was also considered the strongest influence on *likelihood of discharge to residential care*, although by a small margin (35%), followed by dementia (28%), frailty (22%), then delirium (12%).

The perceptions of the influences on *mortality six months post-admission* were more mixed, with the most common selection being frailty (37%), while almost as many (31%) chose DSD, 21% chose delirium and 7% selected dementia. Similarly, the strongest influence on the *likelihood of future admission to residential care within the following year* was viewed as being dementia (34%), followed closely by DSD (30%), then frailty (24%), while delirium was rarely considered the strongest risk (9%). Thus, DSD was considered to have the strongest prognostic significance overall, and dementia was perceived mainly to influence residential care but not mortality.

Respondents were next asked to what degree a delirium episode influences the development (in the coming year) of dementia or frailty, when the person was without that condition at baseline. No age range was specified. Participants (*n* = 279) felt that delirium most strongly influenced the future development of dementia, with 60% saying it had a strong influence and 30% a moderate influence. However, 7% selected a low influence option and 5% were unsure. The responses for delirium influencing future frailty were similar. Nearly half of respondents (49%) felt delirium would strongly influence future frailty, while 40% rated it a moderate influence.

## Discussion

This study describes the perceptions of geriatricians and experienced geriatric trainees (referred to collectively as geriatricians for brevity below) across Europe on the prevalence and co-occurrence of dementia, delirium and frailty, the risk factors for developing delirium, and the influence of delirium on future development of frailty and dementia.

Three quarters of respondents, considering a group of 80-year-old patients within 48 h of an unplanned hospital admission, believed frailty prevalence was 41% or more, whereas the range suggested by 11 studies in a 2022 meta-analysis (MA) with a population age over 80 was 5–68% [26]. Reported frailty prevalence scores are highest using the Frailty Index, followed by Fried’s Phenotype and then the CFS [28]. In our survey, the frailty model/tool considered by the respondent did not overly influence the frailty prevalence.

For dementia prevalence, most respondents estimated a likelihood (21–60%), broadly aligning to the reported estimated range of 26–63% for hospital inpatients of mean age 80 years or older [5]. Delirium was considered more prevalent than dementia, but less prevalent than frailty. Delirium rates in a recent MA for an over 80 cohort (12 studies) were 10–42% [26], whereas almost half of our respondents selected a prevalence of 41–100%.

To explore perceptions of the co-occurrence of conditions (again considering 80-year-olds with an acute admission) the most popular answer was for *people with dementia to also have frailty*, with a third of respondents choosing a prevalence of 61–80% for this scenario. An SR suggests 51–92% of hospitalised older people with dementia are frail [29]. For the converse, i.e. the prevalence of *dementia in older hospital patients with frailty*, most respondents selected categories between 21 and 60%, thus perceiving that people with frailty are less likely to have dementia than a person with dementia having frailty. There is insufficient data on the prevalence of dementia in frail populations in acute hospitals, but frailty is known to increase dementia risk in the community [14, 30].

The most commonly selected option for the percentage of *people with dementia also having delirium* was 41–60%. This aligns with an SA and MA reporting the pooled prevalence of DSD as 48.9% for hospitalised older adults with dementia [31], and previous Irish hospital cohort studies showing 57% DSD prevalence in acute hospitals [32] and 49% in the Emergency Department [33]. However, there was significant variance in opinion. The same prevalence range (41–60%) was selected for *people with delirium also having dementia*, aligning with literature reports of 49–73% of people with delirium having dementia [31–34]. It is well known that dementia and delirium are confused by hospital staff, with delirium incorrectly assumed to be dementia, and delirium missed in people with dementia [35]. It is important that geriatricians are confident advocates of the need to differentiate these, but also mindful of their frequent co-occurrence.

With regard to the prevalence of *frailty in older inpatients with delirium*, the wide spread of responses across categories indicates varied clinical realities across Europe. The most common category was 41–60%. The literature suggests delirium significantly increases the risk of frailty, with 71–92% of older hospital patients with delirium having frailty [16, 29, 36]. Thus, a delirium episode should lead to a comprehensive assessment for frailty risks, alongside a search for delirium precipitants. Equally, the main risks for delirium are advanced age and baseline cognitive vulnerability, particularly MCI and dementia, and neurological conditions such as Parkinson’s disease and stroke, where frailty is common [37, 38]. It should be no surprise that a delirium episode is a marker of frailty, since delirium is precipitated by an illness that perturbs homeostasis, with a much lower threshold in frail older people.

For people *with frailty also having delirium*, most respondents selected the categories within 21–60%. Thus, geriatricians consider frailty to be more prevalent in an older person with delirium than delirium occurring in a frail older person. There are insufficient published data on the prevalence of delirium in frail hospitalised populations, but a 2022 MA suggested a modest RR of 1.66 (1.23–2.22) [26].

Respondents considered severe dementia, and delirium during the last hospital admission, to be the most significant risk factors for an older patient to develop incident delirium. The literature concurs that dementia is a key risk for incident delirium [1, 2]. Interestingly, moderate to severe frailty was considered by respondents to have an almost similar risk to mild dementia, and more risk than MCI. In a dataset across six hospitals in Ireland, 47% of people with mild dementia also had delirium, less than the 78% of people with severe dementia and similar to the 47.5% of people with moderate to severe frailty. In this dataset, only 19.5% of people with MCI had delirium (unpublished data; [32] for dataset details). This concurs with the perception of geriatricians across Europe.

On predisposing risks for delirium, one review described the RR from dementia to be 2.3–4.7 in medical patients and 2.8 in general surgical patients; the RR from “cognitive impairment” was 2.1–2.8 and 3.5–4.2 in these two populations. Previous delirium had a RR of 3.0 in medical patients [8]. This high risk is not surprising, as an episode of delirium indicates delirium vulnerability. Of note, older age had a RR of 4.0 (medical patients) and 3.3–6.6 (surgical patients). Functional impairment had a RR of 2.5–4.0 in these two populations [8]. A 2022 MA found a RR of 1.23–2.22 for delirium in older frail people, with seven of the 26 included studies finding no relationship [26]. Taking these two reviews together, the risk for future delirium seems to descend in order: older age; dementia; previous delirium; cognitive impairment; functional impairment; frailty. Consistent with this, one small hip fracture study found that neither MCI nor frailty increased the risk of delirium, only both together [39].

When asked which of four different conditions (dementia, delirium, DSD, or frailty) had the strongest influence on in-hospital mortality, more than half of respondents chose DSD. This is consistent with a MA showing older inpatients with DSD have a higher mortality risk than patients with dementia alone [31]. A large study of 1,409 geriatric inpatients reported in-hospital mortality rates of 32% for DSD, 29% for delirium and 12% for dementia [40]. More than a third of respondents rated frailty as having the strongest influence on mortality at 6 months post-admission. A geriatric hospital study found six month mortality rates of 7.2% for frail patients [41].

The impact of a delirium episode on future health was considered to have a stronger influence on future dementia (60% chose a “strong influence”) than on future frailty (approximately 50% chose a “strong influence”). Of note, 7% considered delirium’s influence on future dementia to be low. A MA reported in 2021 (4 hip fracture studies, 1 cardiac surgery and 1 geriatric medicine study) found that older inpatients with delirium had an almost twelve times greater probability of developing new dementia compared to inpatients without delirium [42]. A UK population-based study similarly reported that delirium increased the odds of incident dementia almost nine-fold (noting wide 95% confidence intervals: 2.1–35) [3]. Delirium’s influence on future frailty is not reported in the literature, but post-operative delirium increases the probability of functional decline with odds ratio of 2.4 (95% CI, 1.4–4.2) [43].

## Limitations

Although this survey was designed by a team of geriatricians with expertise in dementia, delirium and frailty, and was piloted before dissemination, this work has some limitations. Firstly, participation was on a voluntary basis, and therefore, the relatively small sample of geriatricians who responded might not be representative. Eastern Europe was particularly poorly represented. Although professional networks were used to disseminate the survey, we did not verify the participants’ self-reported inclusion criteria. Another limitation is the use of a survey instrument to draw conclusions across a large region. To reduce the language burden, respondents were offered “tick box” options for the main questions, such as fixed categories for prevalence and co-occurrence data rather than open-ended questions, which limits the resulting analysis. In some European countries, Geriatric Medicine is not a defined speciality. This means responses may have been lower in some countries as potential respondents deemed themselves ineligible. It should also be taken into consideration that some countries are relatively over-represented in the results, including some countries where Geriatric Medicine is not a recognised speciality.

The survey was available in twelve European languages in total, but these were accessible through an embedded link, and it had to be answered in English. Although poor English proficiency would not be a barrier once the translation was at hand, we acknowledge the extra time burden for completion in this way may have been a barrier to response, and not all geriatricians may have been fluent in one of the 12 offered languages. In particular, for respondents in Eastern Europe, a translation was only available in Ukrainian.

## Conclusion

This survey of geriatricians across Europe emphasises the importance of recognising and addressing the interconnected nature of dementia, delirium and frailty in clinical practice. In this survey sample, there was often no consensus on the relative frequency, co-occurrence and consequences of these conditions. There are also discrepancies between the perceptions of the included clinicians (based on their real-life experience) and the reported literature as to the overall prevalence of frailty (and to a lesser extent delirium) in hospitalised older people, the co-occurrence of frailty in people with delirium and the significance of pre-frailty for future delirium events. In some cases, such as frailty concurrent with delirium or dementia, there were little available data in the literature.

These findings highlight the complex reality of co-existing geriatric syndromes in ageing populations and the need for comprehensive, prospective, longitudinal research to better explore these conditions and their interdependencies. This knowledge is necessary to inform guideline and integrated care pathway development for screening and prevention of these conditions and their sequelae. This study serves as a call to action for researchers and funding bodies to facilitate more robust research to ensure geriatricians are well equipped to manage the multifaceted health challenges faced by older adults.

## Supplementary Information

Below is the link to the electronic supplementary material.

Supplementary file1 (DOCX 173 KB)

## Data Availability

The complete survey dataset will be available in Open Science Framework from September 2025 onwards, once all papers are published (the dataset contains other survey elements that will be the subject of future publications by the author group).
