# Supplementary material for: The perceptions of European geriatricians on the co-occurrence and links between dementia, delirium and frailty
Source: Eur Geriatr Med. 2025 Mar 15;16(3):839–49. doi: 10.1007/s41999-025-01173-4 (PMC12174283; doi:10.1007/s41999-025-01173-4)
Supplement: Supplementary file 1 — Supplementary file1 (DOCX 173 KB) [file 41999_2025_1173_MOESM1_ESM.docx]

**The perceptions of European geriatricians on the co-occurrence and links between dementia, delirium and frailty**

Mary Faherty, University College Cork, Centre for Gerontology and Rehabilitation, Cork, Ireland. ORCID 0009-0009-0812-9128. Corresponding author. mfaherty@ucc.ie

Catriona Curtin, University College Cork, Centre for Gerontology and Rehabilitation, Cork, Ireland.

Giuseppe Bellelli, School of Medicina and Surgery, University of Milano-Bicocca and Acute Geriatric Unit, IRCCS Foundation “San Gerardo dei Tintori”, Monza, Italy. ORCID 0000-0001-5430-0947

Enrico Brunetti, Section of Geriatrics, Department of Medical Sciences, University Hospital Città della Salute e della Scienza, Turin, Italy; Department of Experimental and Clinical Medicine, University of Florence, Florence, Italy. ORCID 0000-0003-2028-2319

Mario Bo, Section of Geriatrics, Department of Medical Sciences, University Hospital Città della Salute e della Scienza, Molinette, Turin, Italy

Alessandro Morandi, Department of Clinical and Experimental Science, University of Brescia, Brescia, Italy; Azienda Speciale Cremona Solidale, Cremona, Italy; Vall d’Hebrón Institute of Research, Barcelona, Spain. ORCID 0000-0003-4848-2507

Antonio Cherubini Geriatria, Accettazione Geriatrica e Centro di Ricerca per l'Invecchiamento; IRCCS INRCA, Ancona, Italy; Department of Clinical and Molecular Sciences; Università Politecnica delle Marche, Ancona, Italy.

Massimiliano Fedecostante, Geriatria, Accettazione Geriatrica e Centro di Ricerca per l'Invecchiamento; IRCCS INRCA, Ancona, Italy.

Maria Cristina Ferrara, School of Medicina and Surgery, University of Milano-Bicocca, Milan. ORCID 0000-0002-7971-5943

Alessandra Coin, Geriatrics Unit, Azienda Ospedale – Università Padova, Department of Medicine, University of Padova, Padova, Italy.

Susan D Shenkin, Ageing and Health Research Group, Usher Institute, University of Edinburgh, Scotland, UK. ORCID[0000-0001-7375-4776](https://orcid.org/0000-0001-7375-4776)

Pinar Soysal, Department of Geriatric Medicine, Faculty of Medicine, Bezmialem Vakif University, Istanbul, Turkiye.

Suzanne Timmons, University College Cork, Centre for Gerontology and Rehabilitation, Cork, Ireland. ORCID 0000-0001-7790-9552

Online Resource 1: Supplementary material showing the number of participants by region and country; frailty models/tools most commonly used by respondents; respondents’ assessment of the strength of selected risk factors for developing incident delirium (with data table); respondents’ opinion on whether delirium psychomotor subtypes differ in affecting the risk of developing a future delirium episode and survey questionnaire.

**SUPPLEMENTARY MATERIAL**

**Table 1: No. participants by region and country**

| Region | Country | No. participants |
| --- | --- | --- |
| Eastern Europe (n=20) | Bulgaria | 1 |
|  | Czechia | 2 |
|  | Poland | 8 |
|  | Romania | 8 |
|  | Russian Federation | 1 |
| Western Europe (n=92) | Austria | 5 |
|  | Belgium | 16 |
|  | France | 17 |
|  | Germany | 21 |
|  | Luxembourg | 3 |
|  | Netherlands | 16 |
|  | Switzerland | 14 |
| Northern Europe (n=131) | Denmark | 15 |
|  | Finland | 12 |
|  | Iceland | 4 |
|  | Lithuania | 1 |
|  | Norway | 17 |
|  | Republic of Ireland | 46 |
|  | Sweden | 9 |
|  | UK | 27 |
| Southern Europe (n=137) | Albania | 1 |
|  | Greece | 13 |
|  | Italy | 34 |
|  | Malta | 4 |
|  | North Macedonia | 1 |
|  | Portugal | 20 |
|  | Serbia | 1 |
|  | Slovenia | 1 |
|  | Spain | 33 |
|  | Turkiye | 29 |

**Table 2: Frailty models/tools most commonly used by respondents (multiple answers were allowed, including Other Model/Tool)**

| **Model / Tool** | **No.** |
| --- | --- |
| (Fried) Frailty Phenotype model | 57 |
| (Fried) Frailty Phenotype model + (Rockwood) Cumulative Deficit / FI model | 18 |
| (Fried) Frailty Phenotype model + (Rockwood) Cumulative Deficit / FI model + Clinical Frailty Scale tool | 29 |
| (Fried) Frailty Phenotype model + (Rockwood) Cumulative Deficit / FI model + Clinical Frailty Scale tool + Other Model/Tool | 1 |
| (Fried) Frailty Phenotype model + (Rockwood) Cumulative Deficit / FI model + Other Model/Tool | 2 |
| (Fried) Frailty Phenotype model + Clinical Frailty Scale tool | 36 |
| (Fried) Frailty Phenotype model + Other Model/Tool | 3 |
| (Rockwood) Cumulative Deficit / FI model | 37 |
| (Rockwood) Cumulative Deficit / FI model + Clinical Frailty Scale tool | 55 |
| (Rockwood) Cumulative Deficit / FI model + Clinical Frailty Scale tool + Other Model/Tool | 4 |
| (Rockwood) Cumulative Deficit / FI model + Unsure | 1 |
| Clinical Frailty Scale tool | 90 |
| Clinical Frailty Scale tool + Other Model/Tool | 3 |
| Other Model/Tool | 7 |


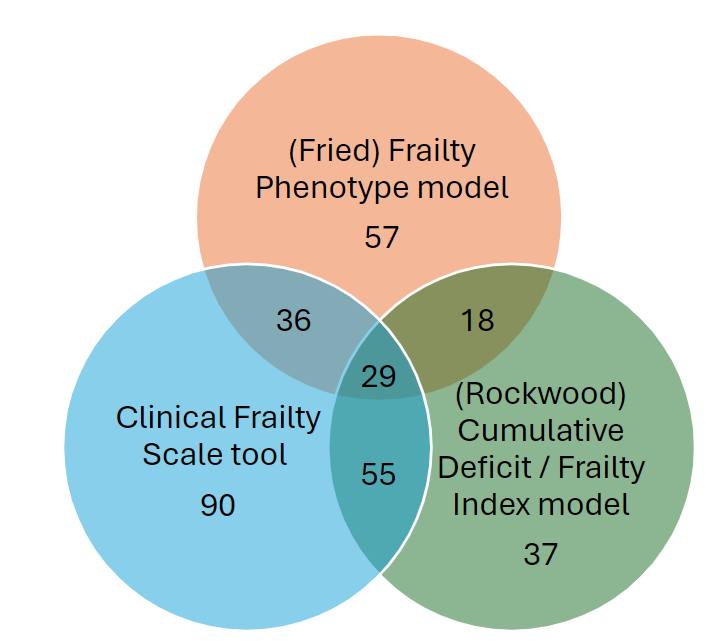


**Figure 1: Venn diagram showing three frailty models/tools commonly used by respondents (multiple answers were allowed)**


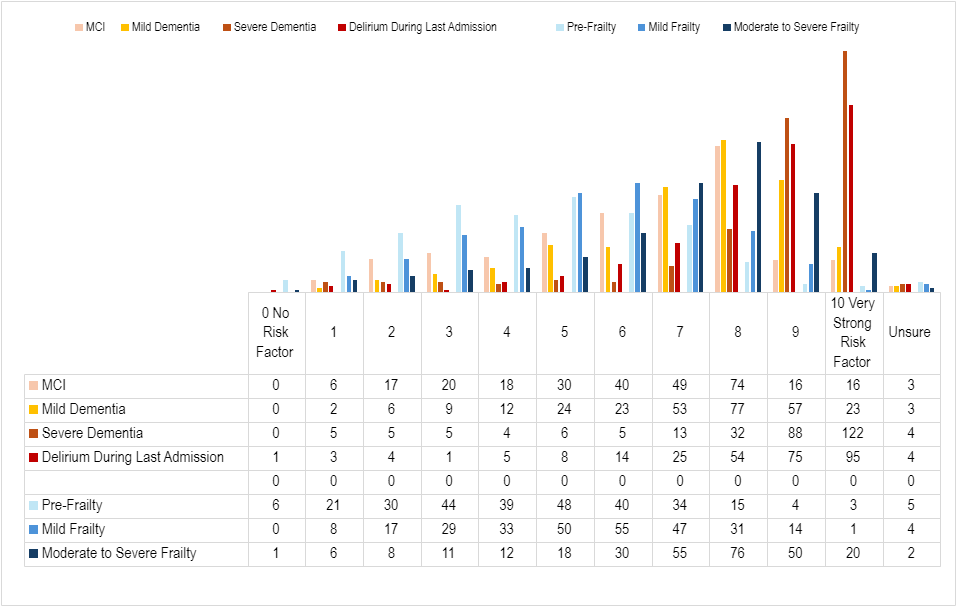


**Figure 2: Respondents’ assessment of the strength of selected risk factors for developing incident delirium, with data table**


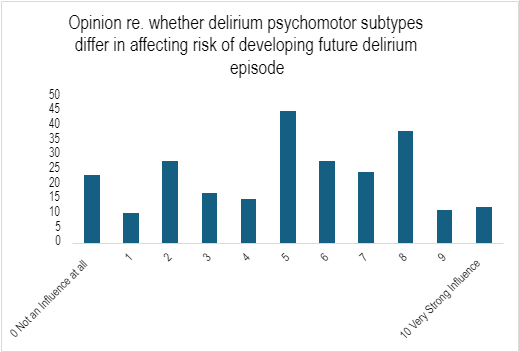


**Figure 3: Respondents’ opinion on whether delirium psychomotor subtypes differ in affecting risk of developing a future delirium episode**

Survey: The relationships between dementia, delirium and frailty as perceived by geriatricians across Europe

**Overview:**Thank you for considering participating in this research project. The following paragraphs explain the project and what your participation would involve, so that you can make an informed choice.

The purpose of this project is to explore the perceptions of geriatricians and senior geriatric trainees in Europe about the complex relationship between dementia, delirium and frailty. This includes relative prevalence, overlaps, causality, etc. The answers you provide will help educators and researchers in all three conditions understand how we perceive these conditions, and particularly where there may be misperceptions or differences in views.

Should you choose to participate, you will be asked to complete a short online, anonymous survey, which will take about 6-12 minutes, depending on your clinical site. Not all questions are mandatory and some important questions come towards the end, so please try to complete the questions at the end before submitting. We have also translated the survey into several European languages if you have difficulty understanding any of the words in the survey. Translations can be accessed by clicking on the following link: **Survey Translations**. Please make sure to answer all survey questions in English only.

Participation in this study is **completely voluntary**. Should you choose to take part, you can skip some questions, or you can decide to end the survey without submitting it. Once you submit your survey, the data cannot be retrieved by us, as it is anonymous.

IP addresses will not be collected at any point, meaning the data you provide cannot be traced back to you. The anonymous data will be stored on the University College Cork OneDrive system and subsequently on a password-protected computer. The pooled data will be stored for a minimum of ten years (as required by our university research data storage policy).

The information you provide may contribute to research publications and/or conference presentations. We do not anticipate any negative outcomes from participating in this study.

This study is unfunded. It has obtained ethical approval from the UCC Social Research Ethics Committee (SREC). If you have any queries about this research, you can contact the postdoctoral researcher, Catriona Curtin, at xxx@ucc.ie. This principal investigator of this survey is Professor Suzanne Timmons.

**Consent:**

If you agree to take part in this survey based on the above information, please indicate your consent below. If you tick ‘yes’, you will be brought to the eligibility check for the survey. Do you consent to participate in this survey?

□ Yes

□ No

**Eligibility:**

Thank you for agreeing to take part in this survey. Please confirm your eligibility by ticking one of the following answers:

□ I am a fully qualified consultant geriatrician (temporary or permanent) working in a European country, currently or within the last 12 months

□ I am a geriatric trainee in my final two years of specialist (higher) geriatric training, based in any European country

□ I am a geriatrician who has retired within the previous three years from working in any European country

□ I do not fit any of the above categories and so I am not eligible for this study

**Section A: Demographics**

Q1. Please select from the following drop down menu, your country of current/recent work.

▼ Albania (1) ... Other (47)

Q2(i). Role: Which of the following relates to you in relation to your current/recent role? Please select one from the following from the drop-down list.

▼ Permanent (1) Temporary (2) Retired (3) Trainee second last year (4) Trainee last year (5)

*Skip To: Q4 If Q2(i). Role: Which of the following relates to you in relation to your current/recent role? Pleas... = Trainee Second Last Year*

*Skip To: Q4 If Q2(i). Role: Which of the following relates to you in relation to your current/recent role? Pleas... = Trainee last year*

Q2(ii). If you are **working or retired**, which of the following is applicable to you in relation to your current or previous (if retired) role. Please select one from the following from the drop-down list:

▼ Fully Clinical (1) Fully Academic (2) Mixed (Both Clinical and Academic Work) (3)

*Display This Question:*

*If Thank you for agreeing to take part in this survey. Please confirm your eligibility by ticking on... != I am a geriatrician who has retired within the previous three years from working in any European country*

Q3. Please select from the following drop-down list, how many years you have worked as a consultant. Please select one response only.

▼ 1-5 Years (1) 6-10 Years (2) 11-15 Years (3) 16-20 Years (4) 21-25 Years (5) 25-30 Years (6) 31-35 Years (7) 35+ Years (8)

Q4. Setting: What is or was the nature of your current or most recent workplace setting(s). Please select from the following all the apply.

□ Acute

□ Community

□ Residential

□ Post-Acute

□ Rehabilitation

□ Outpatient Clinic

□ Academic

Q5. Gender:  What is your gender? Please select one of the following.

▼ Male (1) Female (2) Non-binary /Other (3) Prefer not to say (4)

Q6. Please select your special interests from the following list of options, which are arranged as per the EuGMS Special Interest Groups. (You do not need to be a member of that SIG to select the option). Please select all that apply. [list of EuGMS SIG areas given]

Q7. Have or had (if retired) you a lead role in your setting or region/country in any of the following areas? Please select all that apply. [List of EuGMS SIG areas given]

**Section B: Definitions**

**Dementia** is defined as per DSM-5 criteria. **Delirium** is defined as per DSM-5 criteria. **Delirium superimposed on dementia (DSD)** is where a person with an underlying dementia develops delirium. **Frailty** is understood as a (potentially reversible) clinical syndrome of gradual loss of reserve over time, with increased vulnerability to stressors, leading to functional impairment and adverse health outcomes. The two most common models are:

**The Frailty Phenotype model (Fried / Cardiovascular Health Study)**, wherein certain physical patient characteristics (unintentional weight loss, reduced muscle strength, reduced gait speed, self-reported exhaustion and low energy expenditure) predict poorer outcomes (operationalised as 2 of these = pre-frail; 3+ = frail).

**The Cumulative Deficit / Frailty Index model (Rockwood)**: wherein an accumulation of deficits (ranging from symptoms e.g. loss of hearing or low mood, through signs such as tremor, through to various diseases such as dementia), leads to frailty. In this model, frailty can also be graded in severity (such as in the Clinical Frailty Scale). Derived from the CD/ FI model, the CFS is a hybrid tool relating to both models.

Q1. Please indicate whether, when you consider the term “frailty” in your place of work, you usually use the **Frailty Phenotype** OR **Cumulative Deficit/ Frailty Index**, OR some other conceptualisation, as this will help us to interpret the results: Choose as many as apply.

□ (Fried) Frailty Phenotype model

□ (Rockwood) Cumulative Deficit / Frailty Index model

□ Clinical Frailty Scale tool

□ Unsure

□ Other Model/Tool: Please Give Details

Have you any comment to make so far:

__________________________________________________________________________________

__________________________________________________________________________________

**Section C: Cognitive Frailty**

Without checking in the literature, please answer the following questions:

Q1. Have you heard of the term “cognitive frailty”? Choose one response only from the drop-down list.

▼ I have not heard of this term before (1) I have heard this term before but cannot recall what it is (2) I have heard this term before and might recognize a definition of it (3) I have heard this term before and I am pretty sure I would recognize a definition of it (4) I use this term occasionally in my work (e.g. clinical, research, teaching etc.) (5) I use this term frequently in my work (e.g. clinical, research, teaching etc.) (6) I was involved in defining this term (e.g. a Delphi panel member, etc.) (7)

Q2(i). In your opinion (and again without checking the literature), which of the following best matches “cognitive frailty” as you think it is described in the literature? (regardless of whether you agree with this definition)

□ Subjective cognitive impairment and physical frailty combined

□ Subjective cognitive impairment (regardless of physical status)

□ Mild cognitive impairment and physical frailty combined

□ Mild cognitive impairment (regardless of physical status)

□ Dementia and physical frailty combined

□ Dementia (regardless of physical status)

□ Mild cognitive impairment OR dementia

□ Dementia with previous superimposed delirium

□ Dementia with current superimposed delirium

□ Previous delirium and physical frailty combined

□ Previous delirium (regardless of physical status)

□ Current delirium and physical frailty combined

□ Current delirium (regardless of physical status)

□ Previous delirium and current delirium

□ Current or previous delirium or mild cognitive impairment or dementia

□ Other (please define) __________________________________________________

□ I cannot choose between some of these

Q2(ii) If you were unsure and picked one definition from the above list, what made you choose this option? If you couldn't choose between two options, which two was it and why was this difficult?

__________________________________________________________________________________

__________________________________________________________________________________

Q3(i). The literature defines cognitive frailty as **Mild Cognitive Impairment and physical frailty combined**. To what degree do you agree with this definition?   
                                                                      
**Very Strongly Disagree  (0) ………………………………………………………………………. Very Strongly Agree (10)**
[Scale 0-10. Unsure option also included.]

Q3(ii). Why do you say this?

__________________________________________________________________________________

__________________________________________________________________________________

**Section D: Prevalence and co-occurrence of delirium, dementia and frailty**

*For frailty questions, please answer for whichever frailty model you use or are most familiar with.*

Q1. Considering a group of 80 year old patients, within the first 48 hours after an unplanned admission to acute hospital, what percentage are likely to have:

|  | 0-20% | 21-40% | 41-60% | 61-80% | 81-100% | Unsure |
| --- | --- | --- | --- | --- | --- | --- |
| i) Delirium of any severity |  |  |  |  |  |  |
| ii) Dementia of any severity |  |  |  |  |  |  |
| iii) Frailty of any severity |  |  |  |  |  |  |

Q2. What percentage of people with **dementia** also have:

|  | 0-20% | 21-40% | 41-60% | 61-80% | 81-100% (5) | Unsure (6) |
| --- | --- | --- | --- | --- | --- | --- |
| i) Delirium (i.e., DSD) |  |  |  |  |  |  |
| ii) Frailty |  |  |  |  |  |  |

Q3. What percentage of people with **frailty** also have:

|  | 0-20% | 21-40% | 41-60% | 61-80% | 81-100% (5) | Unsure (6) |
| --- | --- | --- | --- | --- | --- | --- |
| i) Delirium |  |  |  |  |  |  |
| ii) Dementia |  |  |  |  |  |  |

Q4. What percentage of people with **delirium** also have:

|  | 0-20% | 21-40% | 41-60% | 61-80% | 81-100% (5) | Unsure (6) |
| --- | --- | --- | --- | --- | --- | --- |
| i) Dementia |  |  |  |  |  |  |
| ii) Frailty |  |  |  |  |  |  |

Please qualify or clarify any answer if you wish:

__________________________________________________________________________________

__________________________________________________________________________________

**Section E: Risks for Delirium**

Assuming an 80 year old patient is admitted to hospital with a urinary tract infection, to what degree are the following a risk factor for developing **incident delirium?**

|  | 0 | 1 | 2 | 3 | 4 | 5 | 6 | 7 | 8 | 9 | 10 | Unsure |
| --- | --- | --- | --- | --- | --- | --- | --- | --- | --- | --- | --- | --- |
| Mild Cognitive Impairment |  |  |  |  |  |  |  |  |  |  |  |  |
| Mild Dementia |  |  |  |  |  |  |  |  |  |  |  |  |
| Severe Dementia |  |  |  |  |  |  |  |  |  |  |  |  |
| Pre-Frailty |  |  |  |  |  |  |  |  |  |  |  |  |
| Mild Frailty |  |  |  |  |  |  |  |  |  |  |  |  |
| Moderate to Severe Frailty |  |  |  |  |  |  |  |  |  |  |  |  |
| Delirium during last admission |  |  |  |  |  |  |  |  |  |  |  |  |

Please qualify or clarify any answer if you wish:

__________________________________________________________________________________

__________________________________________________________________________________

In your opinion, do delirium psychomotor subtypes (e.g. hypoactive / hyperactive / mixed / no subtype) differ in affecting the risk of developing a future delirium episode?

**Not an influence at all (0) ……………………………………………………………………….Very Strong Influence (10)**[Scale 0-10. Unsure option also]

Please qualify or clarify your answer if you wish:

__________________________________________________________________________________

__________________________________________________________________________________

**Section F: Significance of Delirium, Dementia and Frailty for Prognosis**

*For* ***frailty*** *questions, please answer for whichever frailty model you use or are most familiar with.*Assuming an 80 year old patient admitted to hospital with a urinary tract infection:

Q1. Which condition has the strongest influence on **in-hospital mortality?** *Assume each condition is of moderate severity.*

□ Dementia

□ Delirium

□ Delirium superimposed on dementia (DSD)

□ Frailty

□ Not sure

Q2. Which condition has the strongest influence on **mortality at 6 months post admission?** *Assume each condition is of moderate severity.*

□ Delirium

□ Frailty

□ DSD

□ Dementia

□ Not sure

Q3. Which condition has the strongest influence on **likelihood of discharge (whether directly or via postacute care) to residential care?** *Assume each condition is of moderate severity.*

□ Delirium

□ Dementia

□ DSD

□ Frailty

□ Not sure

Q4. Which condition has the strongest influence on likelihood of future **admission to residential care within the following year?** *Assume each condition is of moderate severity.*

□ Frailty

□ Delirium

□ DSD

□ Dementia

□ Not sure

Q5. For a person **without the condition at baseline**, to what degree does an episode of delirium influence the development, within the coming year, of:

|  | 0 | 1 | 2 | 3 | 4 | 5 | 6 | 7 | 8 | 9 | 10 | Not sure |
| --- | --- | --- | --- | --- | --- | --- | --- | --- | --- | --- | --- | --- |
| Dementia |  |  |  |  |  |  |  |  |  |  |  |  |
| Frailty |  |  |  |  |  |  |  |  |  |  |  |  |
| Functional impairment |  |  |  |  |  |  |  |  |  |  |  |  |

Q6. Please qualify or clarify any answer if you wish:

____________________________________________________________________________________________________________________________________________________________________
